# A method for determining the robustness of bio-molecular oscillator models

**DOI:** 10.1186/1752-0509-3-95

**Published:** 2009-09-21

**Authors:** Reza Ghaemi, Jing Sun, Pablo A Iglesias, Domitilla Del Vecchio

**Affiliations:** 1 Department of Electrical Engineering and Computer Science, University of Michigan, Ann Arbor, USA; 2 Department of Naval Architecture and Marine Engineering, University of Michigan, Ann Arbor, USA; 3 Department of Electrical Engineering and Computer Science, Johns Hopkins, Baltimore, USA

## Abstract

**Background:**

Quantifying the robustness of biochemical models is important both for determining the validity of a natural system model and for designing reliable and robust synthetic biochemical networks. Several tools have been proposed in the literature. Unfortunately, multiparameter robustness analysis suffers from computational limitations.

**Results:**

A novel method for quantifying the robustness of oscillatory behavior to parameter perturbations is presented in this paper. This method relies on the combination of Hopf bifurcation and Routh-Hurwitz stability criterion, which is widely applied in control system design. The proposed method is employed to calculate the robustness of two oscillating biochemical network models previously analyzed in the literature. The robustness bounds here obtained are tighter than what was previously obtained in the literature for both models.

**Conclusion:**

The method here proposed for quantifying the robustness of biochemical oscillator models is computationally less demanding than similar multiparamter variation techniques available in the literature. It also provides tighter bounds on two models previously analyzed in the literature.

## Background

It is well acknowledged that characteristics of biochemical systems, such as oscillatory behavior, are preserved under significantly different environmental conditions, i.e., the systems are robust. Some biological systems have been experimentally proven to be robust [1-3]. Since biological systems are robust, the mathematical models developed to represent their characteristics must also reflect this property. In addition, since it is not possible to precisely determine the parameters in the modeling process, biochemical system models must maintain basic properties, such as oscillatory behavior, in the presence of parameter perturbations [4]. Therefore, robustness is considered as an important measure of the validity of mathematical models. Determining the robustness of the oscillatory behavior of a bio-molecular model has also relevance in design problems. In fact, by determining the region in parameter space where oscillations persist, one can provide guidelines (see [5,6] for example) for designing the components of synthetic oscillators such as those of [7,8].

Parametric robustness of biochemical oscillator networks has been the subject of several studies [4,9]. In these studies, the maximum allowable parameter deviation from nominal values under which the system oscillations persist is considered as a metric for measuring model robustness. Alternative definitions of parametric robustness are proposed in which the sensitivity of the system equilibrium to parameter variation is considered as a measure of robustness [10]. This measure, however, is only applicable to non-oscillatory systems. Bifurcation analysis is employed to study the sensitivity of oscillatory behavior to variations of a single parameter [11-14]. Systems whose robustness to one-parameter-at-a-time variation is established may be sensitive to simultaneous variation of parameters. Unfortunately, analysis of the effect of multiparameter variation on the oscillatory behavior of a system is more computationally demanding compared to one-parameter-at-a-time variation. In particular, systematic variation of multiple parameters suffers from exponential increase in the number of combinations of parameters to be considered. Therefore, multiparametric sensitivity has often been addressed via computer simulations based on Monte Carlo methods [15]. Since this method relies on random variation of all parameters, the resulting robustness evaluation is inconclusive.

Structured Singular Value (SSV) analysis (*μ*-analysis), a tool developed in the field of robust control, has been employed to provide information on the robustness of systems in the presence of multiple and simultaneous parameter variations from nominal values [11]. In this analysis, a parameter called *μ*, is calculated whose inverse determines the maximum allowed parameter variation beyond which the system is destabilized. For oscillatory biochemical networks, destabilization means that the system ceases to oscillate. The advantage of *μ*-analysis over Monte Carlo methods is that, due to its deterministic nature, it can compute the extent of parameter uncertainty for which the model is guaranteed to produce the desired behavior. However, for systems with many parameters, *μ*-analysis is not computationally feasible. Hence, one can only rely on computing upper and lower bounds for *μ*, which determine the maximum stabilizing parameter variation and the minimum destabilizing parameter variation, respectively. To determine whether a system is robust, the lower bound of *μ *must be calculated. However, available algorithms for computing this lower bound suffer from the curse of dimensionality, i.e., computational time grows exponentially with the number of parameters [9]. Another drawback of *μ*-analysis is that it relies on the linearization of the system about the nominal oscillatory trajectory. As a consequence, a specific combination of parameter variations may destabilize the linearized system while still leading to sustained oscillations in the nonlinear system. This is highly likely in biochemical networks as stable periodic solutions often arise due to nonlinear dynamics.

In addition to *μ*-analysis, analysis of robustness of systems to multiparameter variations has been approached by searching the worst case combination of parameter variations that suppresses oscillations. In [9], the integral of the square of the derivative of one of the states, considered as a measure of the occurrence of oscillations, is minimized with respect to parameter variations employing Hybrid Genetic Algorithms (HGA) [16]. This optimization is performed to determine the region in the parameter space where oscillations persist. Since this method is based on exhaustive simulation, it cannot be applied for systems with large numbers of states and parameters due to the associated prohibitive computational cost. In this paper, we introduce a novel robustness analysis method for oscillatory behavior based on the combination of Hopf bifurcation [17] and Routh-Hurwitz stability criterion [18]. Combining these two techniques, we compute a scalar parameter ℛ (encompassing all system parameters), which is solely responsible of Hopf bifurcation. As a consequence, we study the persistence of the periodic orbit as this *single *parameter ℛ is varied. This dramatically reduces the complexity of the problem while retaining the desirable features of multiparameter variation. To translate the maximal variation of ℛ that preserves oscillatory behavior to the maximal variation from a nominal parameter in the original parameter space, we solve an optimization problem. The so obtained maximum parameter variation determines the largest box in parameter space about the nominal parameter values in which the model displays sustained oscillations. Under the assumption that the terms of order higher than three in the Taylor expansion of the system on the center manifold are negligible in the found box, this box provides a tight estimate of the robustness of the system.

We illustrate the application of our approach to models of two molecular networks. The first model describes the molecular network underling adenosine 3',5'-cyclic monophosphate (cAMP) oscillations observed in populations of Dictyostelium cells, proposed by Laub and Loomis in [19]. The second model describes the metabolism of an activated neutrophil granulocyte [20].

We next describe the details of the method proposed in this paper.

## Methods

In this section, we propose a robustness analysis technique based on the combination of Hopf bifurcation [17] and Routh-Hurwitz stability criterion [18]. Consider a model of a biochemical network given as

(1)

in which *x*(*t*) ∈ ℝ^*n *^is a vector whose elements are concentrations of chemical species and *K *∈ ℝ^*p *^is a vector of parameters. Let *K*_0 _∈ ℝ^*p *^be a nominal parameter vector for which system (1) displays a stable periodic orbit. We seek to determine the maximal allowed variation of the parameters *K *from the nominal value *K*_0 _before the stable periodic solution disappears. We tackle this problem by analytically computing the set of all values of *K *at which system (1) admits a Hopf bifurcation. We then compute the largest box in parameter space about *K*_0 _that does not include any of the values of *K *at which the system admits a Hopf bifurcation. Under suitable assumptions, this box is the largest box about *K*_0 _in which sustained oscillations are preserved. This is explained in more detail in the following section.

### Hopf bifurcation analysis

At a Hopf bifurcation, an equilibrium of a dynamical system loses stability as a pair of complex conjugate eigenvalues of the linearization about the equilibrium cross the imaginary axis of the complex plane. We thus first compute the equilibrium of the system as a function of the parameters, i.e., *x*_*e*_(*K*) such that *f*(*x*_*e*_(*K*), *K*) = 0. In systems with S-system representation [10,21], the equilibrium can be calculated analytically. For general systems, we rely on numerical methods such as Newton's iterations to compute *x*_*e*_(*K*) [22]. Once a nominal equilibrium is identified, i.e., *x*_*e*_(*K*_0_), we initialize Newton's method with this nominal equilibrium to calculate the equilibrium when *K *≠ *K*_0_. Then, to determine conditions under which the system undergoes a Hopf bifurcation, we study the behavior of the eigenvalues of the matrix

(2)

Note that even if Hopf bifurcation relies on the properties of the linearization matrix *A*(*K*), it allows to infer the existence of a periodic orbit for the original nonlinear system [17].

Assume there exists an open region *D *⊆ ℝ^*p *^in parameter space about the nominal parameter vector *K*_0 _= (*k*_0, 1_,..., *k*_0, *p*_) and a function ℛ: ℝ^*p *^→ ℝ, called *R*-function, with the following properties:

(i) ℛ(*K*) = 0 if and only if *A*(*K*) has two imaginary eigenvalues while all the others having negative real parts;

(ii) ℛ (*K*) > 0 if and only if *A*(*K*) has two eigenvalues with positive real parts while all the others having negative real parts;

(iii) ℛ(*K*) < 0 if and only if all eigenvalues of *A*(*K*) have negative real parts.

Let *K *= (*k*_1_,..., *k*_*p*_) and assume there exists an open box *B*_δ*_(*K*_0_) = {*K *∈ ℝ^*p*^| |*k*_*i *_- *k*_0, *i*_| <δ* for all *i*} contained in *D *such that

For all *K *∈  (*K*_0_), ℛ(*K*) > 0;

There exists  on the boundary of *B*_δ* _(*K*_0_) such that ℛ() = 0;

There exists a path Γ ending at  in *D*\*B*_δ* _(*K*_0_) such that for all points on the path ℛ(*K*) < 0.

**Figure 1:**
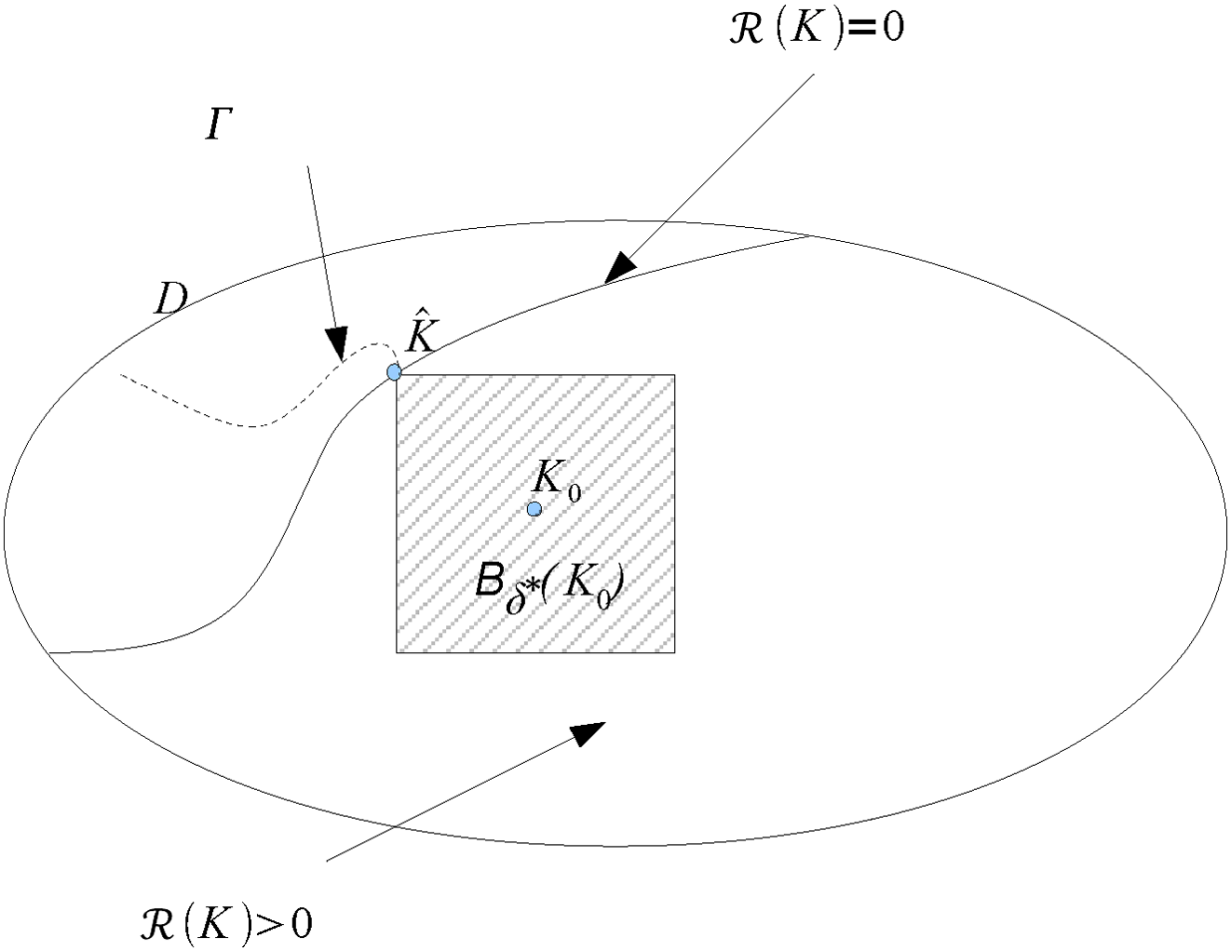
Structure of the box *B*_δ_* (*K*_0_). The box is in the area where ℛ(*K*) > 0 and ℛ vanishes at  on the boundary of the box. The dashed curve shows a path Γ in *D*\*B*_δ_* (*K*_0_) on which ℛ(*K*) < 0.

The sets *D*, *B*_δ* _(*K*_0_), and the path Γ are illustrated in Figure 1.

According to these assumptions, there is a path Γ in parameter space, crossing through , along which ℛ(*K*) is first negative, i.e., all eigenvalues of *A*(*K*) have negative real parts. Then ℛ(*K*) = 0 at *K *= , i.e., *A*() has two imaginary eigenvalues with all the others having negative real parts. Finally, ℛ(*K*) > 0 in *B*_δ* _(*K*_0_), i.e., *A*(*K*) has two eigenvalues with positive real parts with all the others having negative real parts. Therefore, a Hopf bifurcation occurs at , corresponding to ℛ() = 0. A small amplitude periodic orbit appears for small ℛ(*K*) > 0 if the Hopf bifurcation is supercritical. Hence, if ℛ(*K*) is sufficiently small for all *K *∈ *B*_δ* _(*K*_0_) the system admits a stable periodic orbit in *B*_δ* _(*K*_0_) (having ℛ(*K*) small enough in the box *B*_δ* _(*K*_0_) implies that the Taylor expansion of the system on the center manifold passing through  and *x*_*e*_() is such that the terms of order higher than three are negligible in *B*_δ* _(*K*_0_)). According to Hopf bifurcation theorem, at *K *= , the periodic orbit disappears and therefore, we take d* as a robustness measure.

The assumption that ℛ(*K*) is sufficiently small in the open box δ* (*K*_0_) may not hold for all biochemical systems. One can further validate that ℛ(*K*) is sufficiently small in the open box *B*_δ* _(*K*_0_) by simulating the system for ℛ(*K*) between zero and its maximum in *B*_δ* _(*K*_0_) and by verifying that oscillations persist. Since simulation is performed as one scalar parameter ℛ(*K*) varies (as opposed to varying multiple parameters at once), it is computationally feasible. It is worth noting that there may be variations in how the higher order terms in the Taylor expansion grow when ℛ(*K*) grows depending on the path adopted to vary ℛ(*K*) inside the box *B*_δ* _(*K*_0_).

### Determining the R-function ℛ(K)

To construct the function ℛ, we consider the characteristic polynomial of *A*(*K*)

(3)

in which the coefficients of the polynomial are functions of the parameter vector *K*. To evaluate the effect of *K *on the eigenvalues of *A*(*K*), we appeal to the well known Routh-Hurwitz stability criterion [18]. This criterion can be translated into a tabular method, in which for a system with characteristic polynomial *C*(*s*, *K*), the table has *n *+ 1 rows and the following structure depicted in Table 1 (neglecting the dependence on *K*). In this table,  := *a*_2*k*-1_,  := *a*_2(*k*-1)_, *k *= 1, 2, ⋯, ,

**Table 1:** Tabular method for a system with characteristic polynominal C (s, k)

*s*^*n*^	*a*_0_	*a*_2_	*a*_4_	⋯
*s*^*n*-1^	*a*_1_	*a*_3_	*a*_5_	⋯
*s*^*n*-2^				⋯
*s*^*n*-3^				⋯
⋮	⋮	⋮	⋮	⋱
*s*^3^			0	⋯
*s*^2^	^**^		0	⋯
*s*^1^		0	0	⋯
*s*^0^		0	0	⋯

(4)

and

(5)

According to the Routh-Hurwitz criterion, the number of eigenvalues of *A*(*K*) with positive real part is determined by the number of sign changes in the vector

(6)

As shown in Appendix 1, we can take



as the *R*-function.

### Algorithm for calculating the maximum allowed uncertainty δ*

In light of the earlier section, the resulting algorithm for calculating the maximum allowed uncertainty δ* about *K*_0 _for which the stable periodic orbit is preserved is summarized as follows.

#### Algorithm 1

Step 1. Calculate the maximum value of δ*, such that for all *K *∈ *B*_δ* _(*K*_0_), we have that ℛ(*K*) > 0.

Step 2. Identify  on the boundary of *B*_δ* _(*K*_0_) such that ℛ() = 0.

Step 3. Verify that *a*_0_(*K*), *a*_1_(*K*), (*K*, ⋯, (*K*), and (*K*), computed in equation (4), are positive for all *K *∈ *B*_δ* + ϵ _(*K*_0_) for some ϵ > 0.

Step 4. Verify that in an open neighborhood **N**() ⊂ *B*_δ* + ϵ _(*K*_0_) about , we have that ℛ(*K*) ≤ 0 implies  (*K*) is positive. Set *D *:= **N**() ∩ *B*_δ* _(*K*_0_).

Step 5. Verify that the value of ℛ(*K*) for *K *on the path Γ = {(1 -*α*)*K*_0 _+ *α* | *α *> 1} ∩ *B*_δ* + ϵ _(*K*_0_) is negative.

**Remark 1**. If *a*_0_(*K*), *a*_1_(*K*), (*K*, ⋯, (*K*),  and (*K*) are all positive in *D*, Algorithm 1 can be employed with ℛ(*K*) := -(*K*).

Step 5 verifies that ℛ is negative on the path Γ outside the region *B*_δ* _(*K*_0_). Steps 3 and 4 verify that the conditions under which ℛ(*K*) = -*T*(*K*) as given in Appendix 1 are satisfied. In particular, since ℛ(*K*) ≤ 0 is equivalent to *T*(*K*) ≥ 0, Step 4 verifies that condition (b) of Claim 1 in Appendix 1 is satisfied. Step 3 verifies that condition (a) of Claim 1 in Appendix 1 is satisfied. Once the function ℛ(*K*) has been computed, Step 1 can be implemented by calculating the smallest *δ *for which ℛ(*K*) is non-positive on some point in the closed box *B*_*δ *_(*K*_0_). This can be obtained by solving the following optimization problem



This optimization problem provides the value of δ* of Step 1 and the value of  of Step 2. Similarly, Steps 3 and 4 can be implemented by determining the minimum of the designated functions on the specified areas in the parameter space. Step 5 can be implemented through a scalar optimization problem, which can be solved via the bisection method [23].

**Remark 2**. The robustness of the periodic orbit of a system can be evaluated by determining the deviation δ* from the nominal parameter values at which the function ℛ changes sign. This deviation can be numerically computed by employing standard optimization techniques such as SQP and HGA. As a consequence, this technique is computationally lighter than multiparameter robustness analysis based on random-search methods, in which the system is simulated at each point in parameter space which is determined by the random search algorithm.

**Remark 3**. If some key parameters are *a priori *identified to have more significant influence on the system dynamics than others, the proposed algorithm can be employed to analyze the effect of variation of significant parameters while non-significant ones are kept constant at their nominal values. A method for determining the robustness interval specifically for individual parameters is considered in [9].

## Results

In this section, we illustrate the detailed application of Algorithm 1 to two well known models of oscillatory biochemical networks and compare the results with robustness measures previously obtained in the literature.

### Application to the Laub and Loomis model

**Figure 2:**
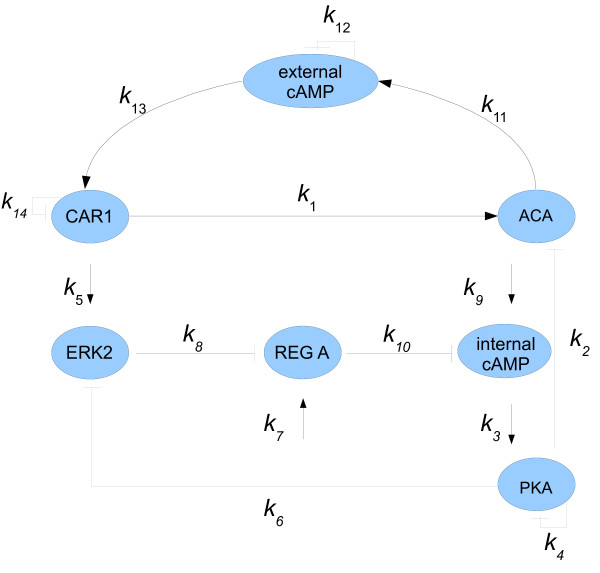
**Laub and Loomis model**.

First, we consider a model of the molecular network underlying adenosine 3',5'-cyclic monophosphate (cAMP) oscillations observed in populations of Dictyostelium cells, proposed by Laub and Loomis in [19]. The model, based on the network depicted in Figure 2, displays spontaneous oscillations in cAMP observed during the early development of Dictyostelium discoideum.

In this model, changes in the enzymatic activities of these proteins are described by the following system of seven non-linear differential equations

(7)

in which the state variable *x *= [*x*_1_, ⋯, *x*_7_] represents the concentration of the seven proteins: *x*_1 _= [ACA], *x*_2 _= [PKA], *x*_3 _= [ERK2], *x*_4 _= [REGA], *x*_5 _= [Internal cAMP], *x*_6 _= [External cAMP] and *x*_7 _= [CAR1]. The fourteen coefficients, *k*_*i*_, *i *= 1, ⋯, 14, are system parameters and we denote *K *= (*k*_1_, ⋯, *k*_14_). It is shown in [19] that oscillations appear at the nominal parameter values given in Table 2. We define the nominal parameter vector *K*_0 _:= [*k*_0, 1_, ⋯, *k*_0, 14_]. To calculate the matrix *A*(*K*) in equation (2), we compute in the next section the equilibrium of system (7).

**Table 2:** Nominal values for each parameter

**Parameter**	**Units**	**Nominal Value**
*k*_0,1_	*min*^-1^	2.0
*k*_0,2_	*Mol*^-1^*min*^-1^	0.9
*k*_0,3_	*min*^-1^	2.5
*k*_0,4_	*min*^-1^	1.5
*k*_0,5_	*min*^-1^	0.6
*k*_0,6_	*Mol*^-1^*min*^-1^	0.8
*k*_0,7_	*Mol*^-1^*min*^-1^	1.0
*k*_0,8_	*Mol*^-1^*min*^-1^	1.3
*k*_0,9_	*min*^-1^	0.3
*k*_0,10_	*Mol*^-1^*min*^-1^	0.8
*k*_0,11_	*min*^-1^	0.7
*k*_0,12_	*min*^-1^	4.9
*k*_0,13_	*min*^-1^	23.0
*k*_0,14_	*min*^-1^	4.5

#### Equilibria of the system

Since the system has S-system structure [10], i.e., the elements of *f*(*x*, *K*) are the addition of two monomials, the equilibrium can be calculated analytically. The equilibrium we consider is given by , in which,



For the detailed derivation, the reader is referred to Appendix 2.

#### Determining the function ℛ

In this section, we employ the explicit representation of the equilibria computed in the previous section to linearize the system and compute the function ℛ. The linearization of the system about the non-zero equilibrium renders the linearization matrix

(8)

The characteristic polynomial of the matrix *A*(*K*) can be written in the following form (neglecting the dependence on *K*)

(9)

in which *a*_*i*_, *i *= 0, ⋯, 6, are calculated analytically as functions of *k*_1_, ⋯, *k*_14_. The corresponding Routh-Hurwitz table is given in Table 3.

**Table 3:** Routh Hurwitz table for characteristic polynomial (9)

s^7^	1	a_2_	a_4_	a_6_
*s*^6^	a_1_	a_3_	a_5_	a_7_
*s*^5^				0
*s*^4^				0
*s*^3^			0	0
*s*^2^			0	0
*s*^1^		0	0	0
*s*^0^		0	0	0

**Figure 3:**
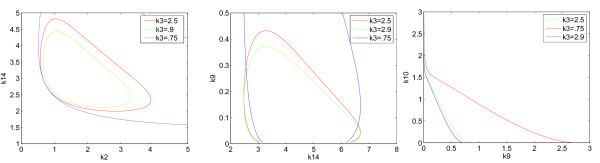
**Zero level sets of the function ℛ in the (*k*_2_, k_14_), (*k*_14_, *k*_9_) and (*k*_9_, *k*_10_) planes for different values of *k*_3_**.

Following Remark 1, we set ℛ(*K*) =  and then show that the values of *a*_1_(*K*),  are all positive in the found set *D*. Since the expression of the function ℛ(*K*) is very long, its exact formula is omitted here. Instead, to provide a qualitative understanding of the behavior of ℛ as a function of the parameter vector *K*, Figure 3 shows the 2-dimensional zero level set of the function ℛ for different nominal values of *K*.

#### Implementation of Algorithm 1

**Step1**. To implement Step 1 of Algorithm 1, we determine the largest *δ *such that ℛ(*K*) is positive in the open box *B*_*δ*_(*K*_0_). The box is defined as follows to reflect the relative variation of parameters instead of absolute change:

(10)

We determine the maximum percentage of the parameter uncertainty under which ℛ(*K*) ≥ 0 via solving the following optimization problem:

(11)

in which Δ = (*δ*_1_, ⋯, *δ*_14_) and *k*_0,1_, ⋯, *k*_0,14 _are the nominal parameters. The above optimization problem determines the smallest *δ *such that the box *B*_*δ*_(*K*_0_) contains an element at which ℛ is non-positive. This is equivalent to determining the largest *δ *such that ℛ is positive inside *B*_*δ*_(*K*_0_). The optimization problem (11) has the following equivalent form:

(12)

where Δ = (*δ*_1_, ⋯, *δ*_14_). This problem is solved numerically employing the Sequential Quadratic Programming (SQP) method [24]. Solving the optimization problem (12) results in δ* = 0.0051 = ||Δ*||∞, where . Although the SQP provides only a local optimal solution, since δ* is a small number, we expect it to be the actual optimal solution. To further verify that the solution is the global optimal, we perform exhaustive search using gridding methods for the points {*k*_0, *i *_- δ*k_0, *i*_, *k*_0, *i*_, *k*_0, *i*_, + δ*k_0, *i*_}. To perform exhaustive search we evaluate ℛ(*K*) for 3^14 ^values of *K *and determine the minimum value. The resulting minimum value is the same as the one obtained with the SQP.

**Step 2**. The solution to the optimization problem (12) provides the optimal values of *δ*_*i*_, *i *= 1, ⋯, 14, i.e., , which make the constraint ℛ(*K*) ≤ 0 active. Therefore, at the point  defined as

(13)

which is on the boundary of the box *B*_δ* _(*K*_0_), we have that ℛ() = 0 and at least for some 1 ≤ *i *≤ 14 || = δ*. The numerical values of the vector  are provided in Table 4. It can be shown that at , with ϵ being sufficiently small, the linearized system around the equilibrium is stable and the system ceases to oscillate.

**Table 4:** Elements of the vector

**Parameter**	**Units**	**Perturbed Value**
	*min*^-1^	1.9898
	*Mol*^-1^*min*^-1^	0.8954
	*min*^-1^	2.5128
	*min*^-1^	1.5076
	*min*^-1^	0.5969
	*Mol*^-1^*min*^-1^	0.8041
	*Mol*^-1^*min*^-1^	1.0051
	*Mol*^-1^*min*^-1^	1.2934
	*min*^-1^	0.3015
	*Mol*^-1^*min*^-1^	0.8041
	*min*^-1^	0.6964
	*min*^-1^	4.9250
	*min*^-1^	22.8827
	*min*^-1^	4.5229

**Steps 3 and 4**. To implement Steps 3 and 4 of Algorithm 1, we consider Remark 1 and evaluate the sign of the elements in the vector  for all *K *in the set *D *defined as *D *:= *B*_δ* + ϵ _(*K*_0_), where ϵ = 0.0001. The sign of these parameters are evaluated via solving the following optimization problem for each function in . This leads to the minimum value that these functions take in *B*_δ* + ϵ_(*K*_0_). Defining  := *a*_1_, this minimum value is provided by

(14)

where Δ = (*δ*_1_, ⋯, *δ*_14_).

The optimization problem is solved for *j *= 0, ⋯, 6 using the gradient descent method. We obtain the minimum values 15.38, 76.13, 184.64, 225.73, 128.74, 93.87 for the functions  and , respectively. Another approach for calculating the minimum value of the functions is performing exhaustive search in the gridded space and we consider the elements

{*k*_0, *i *_- (δ* + ϵ)*δk*_0, *i*_, *k*_0, *i*_, *k*_0, *i *_+ (δ* + ϵ)*k*_0, *i *_*i *= 1, ⋯, 14}. Therefore, to perform exhaustive search we evaluate each of the functions , *j *= 0, ⋯, 6 for 3^14 ^elements (3 elements for each dimension) and determine the minimum value of each function. The resulting minimum values are the same as those obtained employing the gradient descent method.

Since in the box *B*_δ* + ϵ _(*K*_0_) all elements of the vector *v *are positive, the conditions described in Steps 3 and 4 are verified in *D *= *B*_δ* + ϵ _(*K*_0_), according to Remark 1.

**Step 5**. Evaluating ℛ(*K*) for *K *on the path {(1 - *α*)*K*_0 _+ *α* | *α *> 0} ∩ *B*_δ* + ϵ_(*K*_0_), we can show that it is negative except for  at which it is zero and Step 5 of Algorithm 1 is completed.

Therefore, all the Steps in Algorithm 1 are completed and the system has a stable periodic orbit in the open box *B*_δ* _(*K*_0_) with δ* = 0.0051.

To further verify that ℛ is sufficiently small in *B*_δ* _(*K*_0_), we simulate the system for ℛ between zero and its maximum in *B*_δ* _(*K*_0_) and verify that oscillations persist (see Appendix 3 for the details).

### Application to the model of the metabolism of activated neutrophils

The second model we consider for robustness analysis describes the metabolism of an activated neutrophil granulocyte. Neutrophils constitute the pivotal part of the defence system against invading pathogens or bacteria. Upon bacterial invasion, neutrophils leave bloodstream and migrate actively toward the site of infection where they absorb and kill the bacteria. The necessary antibacterial and digestive materials are produced upon activation of the neutrophil. The activation dramatically increases the production of the reduced form of nicotinamide adenine dinucleotide phosphate (NADPH) via hexose monophosphate shunt and initiates the production of the NADPH oxidase complex that assembles at the phagosomal membrane. Electrons are transferred from cytoplasmic NADPH to oxygen on the phagosomal side of the membrane, generating the so-called reactive oxygen species by creating superoxide  as an intermediate step [20,12]. It is shown that in migrating neutrophils the concentration of NADPH and reactive oxygen species oscillate [25,26]. In this paper, we consider the model of the system that is presented by Olsen et al. [20]. The model has 16 states and 24 parameters and it can be written as

(15)

in which the state variable *x *= [*x*_1_, ⋯, *x*_16_] represents the concentration of the 16 species. The factor *ρ *refers to the fractional volume of the phagosome over the cytosol and is assumed to be 0.1. The functions *R*_1_,..., *R*_19 _represent reaction rates and are described in Table 5 along with nominal parameter values.

**Table 5:** Reaction and rate constants of neutrophils

**Reaction**	**Rate expression (*R*_*i*_)**	**Rate constant**
*R*_1_(x)	*k*_1_*x*_5_*x*_1 _- *k*_-1_*x*_2_	*K*_1 _= 5.0 × 10^7^*M*^-^1*s*^-1^
		*k*_-1 _= 58*s*^-1^
*R*_2_(x)	*k*_2_*x*_2_*x*_8_	*k*_2 _= 1.0 × 10^7^*M*^-^1*s*^-1^
*R*_3_(x)	*k*_3_*x*_3_*x*_8_	*k*_3 _= 4.0 × 10^3^*M*^-^1*s*^-1^
*R*_4_(x)	*k*_4_*x*_1_*x*_6_	*k*_4 _= 2.0 × 10^7^*M*^-^1*s*^-1^
*R*_5_(x)		*k*_5 _= 1.0 × 10^7^*M*^-^1*s*^-1^
*R*_6_(x)	*k*_6_*x*_4_*x*_6_	*k*_6 _= 1.0 × 10^5^*M*^-^1*s*^-1^
*R*_7_(x)	*k*_7_*x*_10_*x*_14_	*k*_7 _= 1*M*^-^1*s*^-1^
*R*_8_(x)	*k*_8_*x*_11_*x*_14_	*k*_8 _= 5.0 × 10^7^*M*^-^1*s*^-1^
*R*_9_(x)		*k*_9 _= 5.0 × 10^8^*M*^-^1*s*^-1^
*R*_10_(x)	*k*_10_*x*_16_*x*_10_	*k*_10 _= 1.0 × 10^7^*M*^-^1*s*^-1^
*R*_11_(x)		*k*_11 _= 6.0 × 10^7^*M*^-^1*s*^-1^
*R*_12_(x)	*k*_12_	*k*_12 _= 3.0 × 10^-5^*M*^-^1*s*^-1^
*R*_13_(x)	*k*_13_- *k*_-13*x*14_	*k*_13 _= 1.25 × 10^-5^*M*^-^1*s*^-1^
		*k*_-13 _= 4.5 × 10^-2^*M*^-^1*s*^-1^
*R*_14_(x)	*k*_14_(*x*_7_-*x*_14_)	*k*_14 _= 30*s*^-1^
*R*_15_(x)	*k*_15_(*x*_5_-*x*_12_)	*k*_15 _= 30*s*^-1^
*R*_16_(x)	*k*_16_(*x*_8_-*x*_15_)	*k*_16 _= 10*s*^-1^
*R*_17_(x)	*k*_17_(*x*_9_-*x*_16_)	*k*_17 _= 10*s*^-1^
*R*_18_(x)	*k*_18_(*x*_6_-*x*_13_)	*k*_18 _= 0.01*s*^-1^
*R*_19_(x)		*V *= 288 × 10^-6^*M*^-^1*s*^-1^
		*L *= 550
		*k*_0 _= 1.5 × 10^-6^*M*
		*k*_*N *_= 60 × 10^-6^*M*

In model (15), there are only 14 independent states as  and . Therefore, we let

(16)

Substituting *x*_1 _and *x*_16 _in (15) and employing (16), we have (with abuse of notation) the new state vector *x *= [*x*_2_, ⋯, *x*_15_] with independent states and new differential equation

(17)

in which the parameter vector *K *∈ ℝ^26 ^includes all 24 parameters in Table 5 in addition to *k*_25 _and *k*_26_, which depend on the initial concentration of the molecular species. Assuming initial concentration of 300 *μM *for ferric peroxidase (*x*_1_) and melatonin (*x*_16_) and zero for the rest, we have a nominal value of 300 *μ*M for *k*_25 _and *k*_26 _as in [20]. Moreover, the nominal parameter vector is defined as

(18)

#### Equilibria of the system

The system model does not belong to the category of S-systems. Therefore, we use Newton's method to calculate the equilibrium, *x*_*e*_(*K*), of system (15) by solving *f*(*x*_*e*_, *K*) = 0 for *x*_*e*_. We first simulate the system with nominal parameters *K*_0 _and choose a point on the corresponding periodic orbit to initialize the Newton iterations to calculate *x*_*e*_(*K*_0_). Then for any parameter vector *K *≠ *K*_0_, the Newton's method is initialized by *x*_*e*_(*K*_0_) and the iteration is performed to achieve *x*_*e*_(*K*).

#### Determining the function ℛ

The linearization of the system about the non-zero equilibrium renders the matrix *A *∈ ℝ^14 × 14^, which is calculated numerically as a function of the equilibrium *x*_*e *_and parameter vector *K*. The 14th order characteristic polynomial of the matrix *A *is given (omitting the dependence on *K*) by *C*(*s*) = *s*^14 ^+ *a*_1_*s*^13 ^+ ⋯ + *a*_13_*s *+ *a*_14_. Table 6 shows the Routh-Hurwitz table in which the parameters are functions of *x*_*e *_and *K*. Using Newton's method, *x*_*e *_is calculated as a function of *K *and therefore the vector  is also calculated as a function of *K*. According to equations (5), function ℛ becomes

**Table 6:** Routh Hurwitz table

*s*^14^	1	*a*_2_	*a*_4_	⋯	*a*_14_
*s*^13^	*a*_1_	*a*_3_	*a*_5_	⋯	0
*s*^12^				⋯	
⋮	⋮	⋮	⋮	⋱	⋮
*s*^3^			0	⋯	
*s*^2^			0	⋯	
*s*^1^		0	0	⋯	0
*s*^0^		0	0	⋯	0

(19)

#### Implementation of Algorithm 1

We now apply Algorithm 1 to determine the region in which the stable periodic orbit persists.

**Step 1**. We calculate the maximum value of *δ *such that ℛ is positive in the interior of *B*_*δ*_(*K*_0_), in which

(20)

This problem is equivalent to solving the following optimization problem: *δ *subject to: |*δ*_*i*_| ≤ *δ*, *i *= 1, ⋯, 26 and ℛ(*k*_0,1 _+ *δ*_1_*k*_0,1_, ⋯, *k*_0,26 _+ *δ*_26_*k*_0,26_) ≤ 0. Employing the SQP solver, we achieve δ* ≃ .17. However, employing first Adaptive Search Algorithm [27] and then SQP to the result of the Adaptive Search Algorithm (see Appendix 4 for the details), we achieve δ* = .0591. HGA and then SQP are also employed to calculate δ* which leads to the same results as the algorithm described in Appendix 4.

**Step 2**. The solution to the above optimization problem provides the optimal values for *δ*_*i*_, *i *= 1, ⋯, 14, i.e.,  that make the constraint ℛ(*K*) ≥ 0 active. Therefore, at the point  defined as  in which [*δ*_1_, ⋯, *δ*_26_] = 0.0591 [-1, 1, -1, -1, 1, -1, -1, 1, 1, -1, 1, -1, 1, -1, 1, -1, -1, -1, -1, -1, 1, -1, 1, -1, 0, 0], which is on the boundary of the box *B*_δ* _(*K*_0_), ℛ vanishes, i.e., ℛ() = 0. It can be shown that at the point , with ϵ being sufficiently small, the linearized system about the equilibrium is stable and the system ceases to oscillate.

**Step 3**. Let us consider the box *B*_δ* + ϵ_(*K*_0_) for ϵ = 0.0001. Then, in *B*_δ* + ϵ_(*K*_0_) the functions  and  are positive if their minimum in the box is positive. We determine the minimum of each of the functions in the box *B*_δ* + ϵ_(*K*_0_) employing a HGA and SQP. The minimum value of the functions  and  are found to be 172.91, 8214.8, 173070, 1.8478*e *+ 006, 1.0279*e *+ 007, 2.7101*e *+ 007, 3.6251*e *+ 007, 2.6068*e *+ 007, 1.0073*e *+ 007, 1.7745*e *+ 006, 1.3174*e *+ 004, and 5.1522*e *- 004, respectively.

**Step 4**. By defining a small neighborhood about , that is, *B*_.001_(), we obtain that (*K*) is positive in *B*_.001_() and therefore Step 4 of Algorithm 1 is completed with **N**() := *B*_.001_() and *D *:= **N**() ∪ *B*_δ* _(*K*_0_).

**Step 5**. We evaluate ℛ(*K*) for *K *on the path Γ = {(1 - *αK*_0_) + *α* | *α *> 0} ∩ *B*_δ* + ϵ _(*K*_0_) and confirm that it is negative except on  where ℛ is zero. Therefore, Step 5 of Algorithm 1 is completed and the largest box about *K*_0 _in which the system has a stable periodic orbit is *B*_δ* _(*K*_0_) with δ* = 0.0591, provided that ℛ is sufficiently small on *B*_δ* _(*K*_0_).

To verify that ℛ is sufficiently small in the box *B*_δ* _(*K*_0_), we simulate the system for ℛ ranging from zero to the maximum attained in *B*_δ* _(*K*_0_) and verify that oscillations persist (see Appendix 5 for the details).

## Discussion

The method introduced in this paper relies on the computation of ℛ, that is, the *scalar *function of system parameters whose sign determines the existence of the stable periodic orbit. The robustness of the periodic orbit of a system can be evaluated by determining the deviation δ* from the nominal parameter values at which the function ℛ changes sign. This deviation can be numerically computed by employing standard optimization techniques such as SQP and HGA. As a consequence, this technique is computationally lighter than multiparameter robustness analysis based on random-search methods, in which the system is simulated at each point in parameter space. Nevertheless, since ℛ encompasses all system parameters, this method retains the desirable features of multiparameter robustness analysis. Moreover, the proposed method provides more accurate robustness measures when compared to methods based on the linear approximation of the system about the nominal periodic orbit, as it is shown in the first application example. The proposed method relies on Hopf bifurcation theorem and on the assumption that ℛ(*K*) is sufficiently small for *K *inside the box *B*_δ* _(*K*_0_). This guarantees that the terms of order higher than three in the Taylor expansion of the system on the center manifold about  and *x*_*e*_() are negligible in the box *B*_δ* _(*K*_0_). To provide evidence that this assumption is satisfied, we vary ℛ from its minimum to its maximum in the box *B*_δ* _(*K*_0_) and verify via simulation that the periodic orbit persists. This simulation step does not present computational limitations. It is in fact performed as one *scalar *parameter (ℛ) is varied as opposed to varying multiple parameters at once as in multiparameter robustness analysis. The underlying assumption for the approach presented in this paper to provide a tight bound on the robustness of the system is that the nominal periodic orbit of the system originates from a Hopf bifurcation. If this were not the case, the provided bound would not necessarily be meaningful as other types of bifurcations may be responsible of the birth and death of the periodic orbit. Therefore, the approach of this paper is generally applicable and restricted to those natural oscillatory systems exhibiting Hopf bifurcation.

For the Laub and Loomis model, previous work employing HGA, in which the system is simulated at each point in parameter space, the robustness of the system was determined as 0.6% [9]. Employing the method proposed in this paper, the robustness of the system has been determined as δ* = 0.51%. This bound is tight, as the system ceases to oscillate at a combination of parameters that is away from the nominal value *K*_0 _only slightly more than δ*. This bound is therefore tighter than the one found employing *μ *analysis or global/hybrid optimization methods.

For the model of the oscillatory metabolism of activated neutrophils, previous work only performed one-parameter-at-a-time variation [12]. According to this single parameter variation analysis, the minimum deviation from nominal values of parameters which causes the periodic orbit to disappear is 16.67%. By contrast, employing the method proposed in this paper, the robustness of the system has been quantified as δ* = 5.91%. This bound is tight as a combination of parameters that is away from the nominal point *K*_0 _slightly more than δ* has been determined at which the system ceases to oscillate. This result shows that the oscillatory behavior of this model is not as robust with respect to parameter variation as it was perceived.

## Conclusion

The robustness analysis of bio-molecular systems is an important problem in systems and synthetic biology. Previously, the robustness of a system with respect to parameter variations has been investigated by employing *μ *analysis on the linearized system about nominal periodic orbit or by applying HGA, in which the system is simulated for each combination of parameter values. In this paper, a method based on the combined application of Hopf bifurcation and Routh-Hurwitz stability criterion is introduced. We computed a *scalar *function of all system parameters whose sign determines the existence of a stable periodic orbit. This method is applied to two bio-molecular systems: the Laub and Loomis model and the model of the oscillatory metabolism of activated neutrophils. The maximum allowed parameter variation with respect to nominal values under which the system preserves oscillatory behavior is calculated. For the Laub and Loomis model, the computed maximum allowed variation is tighter than what was obtained with previous multiparametric analysis methods. For the model of activated neutrophils, only single parameter variations were considered in the literature to evaluate parametric robustness. Employing the method proposed in this paper, we evaluate the robustness of the system to be about one third of the one estimated in the literature employing single parameter variations.

## Authors' contributions

RG developed the computational techniques outlined in the paper, applied the techniques to the two systems described in the paper, and contributed to the writing of the paper. PI contributed to interpreting the results of the analysis on the first model, suggested the second model as one for which the proposed techniques would apply, and contributed to interpreting the results of the analysis on the second model. JS contributed to the writing and organization of the paper, and helped to enhance the numerical robustness analysis tools used in this paper. DDV developed the theoretical framework on which the proposed computational techniques are based and lead the writing and organization of the paper. All authors read and approved the final manuscript.

## Appendix

### Appendix 1

The following result gives conditions for which -*T*(*K*) can be taken as an *R*-function.

**Claim 1**. *Assume that for all K *∈ *D*,

*(a) a*_0_(*K*), *a*_1_(*K*), (*K*), ⋯, (*K*) *and *(*K*) *are positive;*

*(b) K *∈ *D *∩ {*K *| *T*(*K*) ≥ 0} *implies that *(*K*) > 0.

*Then *ℛ(*K*) = -*T*(*K*) *is an R-function*.

*Proof*. If *T*(*K*) = (*K*)(*K*) < 0 for *K *∈ *D*, then when *K *∈ *B*_δ* _(*K*_0_) either (*K*) is positive and (*K*) is negative or *viceversa*. Therefore, given (a), there are always two changes of sign in the vector *v*(*K*) in (6) for all *K *∈ *D*. Hence, according to Routh-Hurwitz criterion, there are two eigenvalues with positive real part while all the others have negative real part in *D*. Therefore, ℛ(*K*) = -*T*(*K*) satisfies property (ii). According to condition (b), *T*(*K*) = 0 implies (*K*) > 0 and (*K*) = 0. Therefore, from the Routh-Hurwitz criterion, there are two imaginary eigenvalues and all the others have negative real part. As a consequence, ℛ(*K*) = -*T*(*K*) satisfies property (i).

According to (b), if *T*(*K*) > 0 when *K *∈ *D*, then (*K*) > 0. This, along with condition (a), implies by Routh Hurwitz criterion that all eigenvalues of *A*(*K*) have negative real parts. Consequently, ℛ(*K*) = -*T*(*K*) satisfies property (iii).

### Appendix 2

Let us denote by  an equilibrium of the system, that is, *f*(*x*_*e*_(*K*), *K*) = 0. From the 7th and 6th differential equations of the model in equation (7), we have

(21)

and therefore

(22)

Substituting expression (22) in the first differential equation of (7), we have the following result

(23)

or

(24)

Equation (23) and *f*(*x*_*e*_(*K*), *K*) = 0 imply that

(25)

For the nonzero equilibrium, from equation (24) we have that

(26)

From the 3rd differential equation of (7), equation (22) and equation (24), we obtain that

(27)

From the 5th differential equation of (7) and equation (26), we have that

(28)

Substituting equations (27) and (28) in the 4th differential equation of the equation (7), we have that

(29)

Employing equation (29) in equations (21), (22), (27) and (28) we have that

(30)

(31)

(32)

(33)

Therefore, the system has two equilibrium points. One is determined by equation (25), which we do not consider for Hopf bifurcation, because negative values for the states *x*_1_, ⋯, *x*_7 _would result from oscillations and protein concentration is always positive. The other non-zero equilibrium, employed next, is  determined by equations (24-26-29-30-31-32-33).

### Appendix 3

In order to perform this one-dimensional parameter study, we define a path in parameter space along which ℛ changes from zero to its maximum value in the box *B*_δ* _(*K*_0_) monotonically. In the following, we show that ℛ is monotonically increasing with respect to parameter *k*_1 _in *B*_δ* _(*K*_0_). Therefore, the path is created by varying *k*_1 _from zero at  to the point at which ℛ reaches its maximum value, while keeping all other parameters constant to the values taken at .

To check the monotonicity of the function ℛ with respect to *k*_1 _over the box *B*_δ* _(*K*_0_), we first define a new function *H *as follows

(34)

in which *K *= [*k*_1_, ⋯, *k*_14_]. Then, we define the following optimization problem

(35)

in which Δ = [*δ*_1_, ⋯, *δ*_14_]. Employing the gradient descent method, we obtain the maximum value of *H*(*K*) over the box *B*_δ* _(*K*_0_) as -24.9152. Therefore,  ℛ(*K*) is positive over the box *B*_δ* _(*K*_0_), which implies that ℛ is monotonically increasing with respect to *k*_1_.

The maximum value of ℛ in the box *B*_δ* _(*K*_0_) is computed by solving the following optimization problem

(36)

where Δ = [*δ*_1_, ⋯, *δ*_14_]. This problem is solved again through the gradient decent method. Denoting  the solution of (36), we have that  = 4.9828. By varying parameter *k*_1_, we thus simulate the system for ℛ ∈ [0, ]. Simulation results show that the system has a periodic orbit for 0 < ℛ≤ .

### Appendix 4

Consider the optimization problem *δ *subject to: |*δ*_*i*_| ≤ *δ*, *i *= 1, ⋯, 26 and ℛ(*k*_0,1 _+ *δ*_1_*k*_0,1_, ⋯, *k*_0,26 _+ *δ*_26_*k*_0,26_) ≤ 0. We adopt the following solution:

1 Set *δ*^*l *^= 0.17, *l *= 1, *K*_*init *_= *K*_0_.

2 We consider the following optimization problem



We solve this optimization problem using the direct search method of the Genetic Algorithm toolbox of Matlab, with mesh adaptive search algorithm [27], using the initial guess *K*_*init*_. The solution is taken as an initial guess for SQP and the solution provided by SQP is considered as the solution of the above optimization problem. If at the optimal point , ℛ(*K*^*l*^) is non-negative, terminate.

3 Determine 0 <*α *≤ 1 such that ℛ(*αK*_0 _+ (1 - *α*)*K*^*l*^) = 0 (using methods such as bisection [23]).

4 Set *K*_*init *_= *αK*_0 _+ (1 -*α*)*K*^*l*^, *l *= *l *+ 1 and .

5 Go to 2.

### Appendix 5

**Figure 4:**
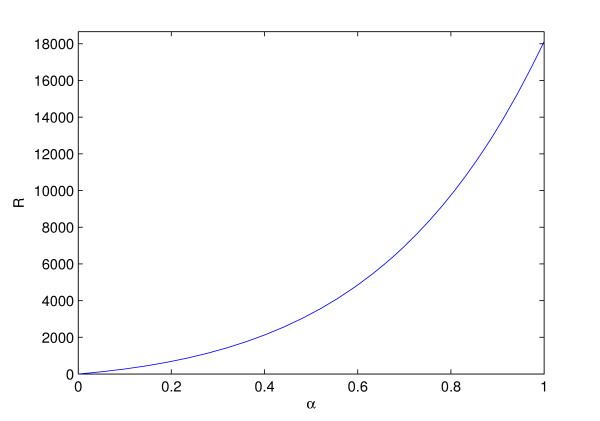
**Variation of function ℛ(*K*) with *α***.

In order to perform this one-dimensional parameter study, we define a path on which ℛ changes from zero to its maximum value in the box *B*_δ* _(*K*_0_) monotonically. Therefore, we first determine the maximum value of ℛ in the box *B*_δ* _(*K*_0_) by employing HGA and SQP. The maximum value of ℛ is achieved at  = *k*_0,1 _+ *δ*_1_*k*_0,1_, ⋯, *k*_0,26 _+ *δ*_26_*k*_0,26 _with



and it is 18665. We vary the value of ℛ from zero at  to its maximum at  by varying *α *from zero to one in (1 - *α*) + *α*. Figure 4 shows the variation of ℛ with respect to *α*.

In order to simulate the system over different values of ℛ, we vary the value of *α *from 0 to 1 at 855 points such that ℛvaries of at most 20 at each step. The simulation results show that the system has a periodic orbit over the interval 0 < ℛ < 18665.

## References

[B1] Barkai N, Leibler S (1997). "Robustness in simple biochemical networks". Nature.

[B2] Alon U, Surette M, Barkai N, Leibler S (1999). "Robustness in bacterial chemotaxis". Nature.

[B3] von Dassow G, Meir E, Munro EM, Odell GM (2000). "The segment polarity network is a robust development module". Nature.

[B4] Savageau MA (1971). " Parameter sensitivity as a criterion for evaluating and comparing the performance of biochemical systems". Nature.

[B5] Del Vecchio D (2007). "Design and Analysis of an Activator-Repressor Clock in *E. coli*". Proceedings of American Control Conference, New York.

[B6] El Samad H, Del Vecchio D, Khammash M (2005). "Repressilators and Promotilators: Loop Dynamics in Gene Regulatory Networks". Proceedings of American Control Conference, Santa Barbara.

[B7] Elowitz MB, Leibler S (2000). "A Synthetic Oscillatory Network of Transcriptional Regulators". Nature.

[B8] Atkinson M, Savageau M, Myers J, Ninfa A "Development of Genetic Circuitry Exhibiting Toggle Switch or Oscillatory Behavior in *Escherichia coli*". Cell.

[B9] Kim J, Bates DG, Postlethwaite I, Ma L, Iglesias PA (2006). "Robustness analysis of biochemical network models". IEE Proceedings Systems Biology.

[B10] Chen BS, Wang YC, Wu WS, Li WH (2005). "A new measure of the robustness of biochemical networks". Bioinformatics.

[B11] Ma L, Iglesias PA (2002). "Quantifying robustnes of biochemical network models". BMC Bioinformatics.

[B12] Jacobson EW, Cedersund G (2008). "Structural robustness of biochemical network models-with application to the oscillatory metabolism of activated neutrophils". IET Systems Biology.

[B13] Wolf J, Becker-Weiman S, Heinrich R (2005). "Analysing the robustness of cellular rhythms". IEE Proceedings Systems Biology.

[B14] Stelling J, Gilles ED, Doyle FJ (3215). "Robustness properties of circadian clock architectures". Proc Natl Acad Sci USA.

[B15] Liu JS (2001). "Monte Carlo strategies in scientific computing".

[B16] Lobo FG, Goldberg DL (1996). "Decision making in a hybrid genetic algorithm".

[B17] Wiggins S (2003). "Introduction to Applied Nonlinear Dynamical Systems and Chaos".

[B18] Hurwitz A (1964). "On the conditions under which an equation has only roots with negative real parts". Selected Papers on Mathematical Trends in Control Theory, New York, Dover.

[B19] Laub MT, Loomis WF (1998). "A molecular network that produces spantaneous oscillations in excitable cells of Dictyostelium". Mol Biol Cell.

[B20] Olsen LF, Kummer U, Kindzelskii AL, Petty HR (2003). "A model of oscillatory metabolism of activated neutrophils". Biophysical Journal.

[B21] Voit EO (2000). "Computational Analysis of Biochemical System".

[B22] Kelley CT (2003). "Solving Nonlinear Equations with Newton's Method". Fundamentals of Algorithms, SIAM, Philadelphia.

[B23] Burden RL, Faires JD (2000). "Numerical Analysis".

[B24] Flecher R (1981). "Practical methods of Optimization".

[B25] Petty RH (2000). "Neutrophil oscillations: temporal and spatiotemporal aspects of cell behaviour". Immunol Res.

[B26] Amit A, Kindzelskii AL, Zanont J, Jarvis JN, Petty RH (1999). "Complement deposition of immune complexes reduces the frequencies of metabolic, proteolytic and superoxide oscillations of migrating neutrophils". Cell Immunol.

[B27] Michael LR, Torczon V (2000). "Pattern Search Methods for Linearly Constrained Minimization". SIAM Journal on Optimization.

